# Developing an Exercise Attitudes and Behavior Intentions Questionnaire for Survivors of Aortic Dissection: An Exploratory Factor Analysis

**DOI:** 10.31083/j.rcm2310337

**Published:** 2022-10-11

**Authors:** Danni Feng, Sufang Huang, Xiaorong Lang, Yuchen Liu, Kexin Zhang

**Affiliations:** ^1^Tongji Hospital, Tongji Medical College, Huazhong University of Science and Technology, 430030 Wuhan, Hubei, China; ^2^School of Nursing, Tongji Medical College, Huazhong University of Science and Technology, 430030 Wuhan, Hubei, China

**Keywords:** aortic dissection, exercise, attitudes, behavior intentions, reliability, validity

## Abstract

**Purpose:**

Our study aimed to develop a questionnaire to assess the reliability and validity of exercise attitudes and behavior intentions among survivors of an aortic dissection (AD).

**Methods:**

There were two phases to the study between April 2021 and April 2022. Phase I involved the development of an initial version of the Exercise Attitudes and Behavior Intentions Questionnaire (EABIQ) through literature reviews, qualitative interviews, Delphi expert consultations and a pre-experimental study. During Phase II, the reliability and validity of the questionnaire was assessed in 160 survivors with AD.

**Results:**

A 62-item EABIQ for AD survivors was developed. Eleven common components with eigenvalues larger than 1 were identified by exploratory factor analysis. The scale’s variance explained cumulatively rate was 75.216%. The content validity index at the item level for the EABIQ varied from 0.813 to 1.000 and the S-CVI/Ave was 0.934. The correlation coefficients between each scale dimension and the overall scale ranged from 0.405 to 0.785, with all *p*-values less than 0.05. Cronbach’s alpha for the whole scale was 0.929, with Cronbach’s alpha for each domain ranging from 0.835 to 0.965. The overall scale split-half reliability coefficient was 0.960, with each domain’s split-half reliability coefficient ranging from 0.844 to 0.962.

**Conclusions:**

The AD exercise attitudes and behavior intentions questionnaire has high reliability and validity and is generally consistent with the hypothetical theoretical framework. It can be used as a judgment tool to measure the exercise behavior for AD patients.

## 1. Introduction

Regular physical exercise is a part of a healthy lifestyle and is beneficial to physical and mental health. Many studies have shown that exercise can improve blood glucose, blood lipid and blood pressure, and reduce the impact of depression and stress on the human body, which leads to a healthy lifestyle and improved quality of life [1, 2, 3, 4, 5]. Moderate physical activity can also reduce the incidence of cardiovascular disease and adverse heart events [6, 7], and can lead to a substantial drop in all-cause mortality [8]. Exercise-based cardiac rehabilitation has been shown to improve outcomes in patients suffering from cardiovascular disorders such as coronary heart disease, atrial fibrillation, heart failure, and the need for cardiac resynchronization treatment [9, 10, 11, 12]. Aortic dissection (AD) is a pathological condition in which blood from an aortic intimal tear enters the aortic media and extends down the long axis of the aorta, resulting in the formation of true and false aortic lumens. Previous research has demonstrated that regular exercise is both safe and useful for AD patients [13, 14, 15, 16, 17]. Patients with AD are advised to perform light to moderate aerobic exercise (3-5METs) for at least 30 minutes for a total of 150 minutes/week to reduce resting blood pressure [18]. Competitive sports and isometric heavy weightlifting should be discouraged [18]. However, patients with AD may not exercise because of pain, fear of accidents during exercise, worries about disease recurrence, and other reasons [19]. Thus, it is very important to understand the attitudes and intentions of AD survivors before the formation of specific exercise programs and guidelines. Exercise attitude refers to the individual’s cognition, emotion and behavior tendency to exercise behavior, and the degree of positive or negative evaluation of behavior performance [20, 21]. Behavior intention is defined as an individual’s tendency to take exercise behavior. It is a cognitive activity that reflects an individual’s willingness and conscious plan to engage in the behavior [22, 23]. There is a link between attitude and behavior. Previous studies have shown that attitudes and beliefs are essential for individuals to accept and adhere to exercise [24, 25]. In order to promote positive health behavior and control health risk behavior, health psychologists have developed several relevant theoretical models of health behavior change, including the continuous theoretical model and the stage theoretical model. In 1992, Schwarzer proposed the Health Action Process Approach (HAPA) hypothesis [26]. This theory integrates the relevant concepts in the continuous theoretical model and the stage theoretical model. In HAPA, healthy behavior change is viewed as a continuous change process that includes the initiation, maintenance, and recovery of behavior. The theoretical model is built around behavior intention as an essential determinant of health behavior. The goal of the study is to create a questionnaire according to the HAPA theory to evaluate the attitudes and behavior intentions of AD patients about exercise, as well as to verify its reliability and validity, so as to provide a basis for personalized phased intervention programs in the future, boost patient rehabilitation, and increase post-operative quality of life.

## 2. Methods

There were two phases to the study between April 2021 and April 2022: (1) focusing on the development of Exercise Attitudes and Behavior Intentions Questionnaire (EABIQ) to create a pool of items (Phase I) and (2) testing the reliability and validity of the EABIQ (Phase II).

### 2.1 Phase I: Questionnaire Development

#### 2.1.1 Item Generation

We used the theory of HAPA to guide the development of the tool’s measurement properties [26]. It is divided into two processes: (1) motivation phase including action self-efficacy, outcome expectations, and risk perceptions and (2) volition phase including action planning, coping planning, maintenance self-efficacy, and recovery self-efficacy.

Twenty-four AD patients in Wuhan, China were invited to participate in semi-structured interviews from April 2021 to June 2021 [19]. In addition, we added a dimension of perceived social support according to the semi-structured interviews. Based on the literature review, an initialized entry pool with 83 items was formed by analyzing the information, with a 5-point Likert scale ranging from ‘1’ (complete disagreement) to ‘5’ (complete agreement).

#### 2.1.2 Delphi Expert Consultations 

Two rounds of Delphi expert consultations, involving experts in the areas of cardiovascular surgery, cardiology, rehabilitation, psychology, were carried out in order to examine the 83-item version’s content validity of the EABIQ. The number of Delphi expert consultation (5–20) was sufficient [27]. Therefore, in the initial round, 18 experts were asked to demonstrate and modify the entry pool by email. Experts were requested to rate the importance of each issue on a scale from 1 (not important) to 5 (very important). We calculated the importance mean and the coefficient of variation (CV, i.e., the standard deviation of each item divided by the mean). Items with the mean of importance ≥3.5 and CV ≤0.3 were included [28], other items that did not meet the conditions were deleted or modified according to expert opinions. The Kendall coefficient W test was used to assess the panel of experts’ consensus on agreement [29]. The degree of expert authority coefficient (Cr) is often determined by experts’ judgment (Ca) and experts’ familiarity (Cs) [30]. The four components of the judgment base are as follows: practical experience, theoretical analysis, references to domestic and international literature, and intuitive emotion. Expert familiarity with problems is stated in five levels: highly familiar (0.9), somewhat familiar (0.7), generally familiar (0.5), less familiar (0.3), and unfamiliar (0). The second round of expert consultation would occur when experts gave new opinions. Sixty-six items were maintained for phase 2 after two rounds of consultation and incorporation of their suggestions.

#### 2.1.3 Pre-Experimental Study

A pre-experimental study was then performed in 20 individuals with various kinds of AD utilizing the EABIQ 66-item version to investigate the clarity and application of the EABIQ items. Following the pre-experimental study, no items were deleted or revised. Patient replies were simple to comprehend and did not take much time to complete (about 15–20 min). To avoid the influence of repeated replies on the results, patients in the pre-experimental study were omitted from the validity and reliability tests.

### 2.2 Phase II: Reliability and Validity

#### 2.2.1 Setting and Study Participants

A descriptive cross-sectional exploratory investigation was carried out between November 2021 and April 2022 from the Cardio-Vascular Surgery Department of Wuhan, China’s third-class Class A hospital. The method of convenient sampling was employed. More than 150 sample sizes are sufficient for exploratory factor analysis according to Guadagnoli and Velicer [31]. The questionnaire was completed by 160 AD patients who satisfied the inclusion criteria. Inclusion criteria included: (1) patients with AD diagnosed by CT; (2) age ≥18 years; (3) stable condition, stable vital signs, no serious complications; (4) voluntary participation; and (5) patients who can understand and communicate in language or words were included in the study. Those with psychiatric problems, cognitive abnormalities, or who were comatose were excluded, as were patients who were still in an unstable condition.

#### 2.2.2 Item Analysis

In this study, the following four methods were combined to screen items [32]: Critical ratio method, correlation method, reliability test, commonalities and factor loadings. Items were eliminated if they failed to fulfill three of the six indices: (1) critical ration (CR) ≥3.00; (2) coefficient of item-to-total correlation (r) ≥0.40; (3) correlation coefficient between changed item and total score ≥0.40; (4) Cronbach’s coefficient after item deletion would be no greater than the entire scale’s internal consistency value; (5) the common value ≥0.20; (6) factor loading ≥0.45.

#### 2.2.3 Exploratory Factor Analysis (EFA) for Construct Validity Assessment

To determine if the items on this questionnaire were appropriate for EFA, the Kaiser-Meyer-Olkin (KMO) measure and Bartlett’s test of sphericity were computed. KMO >0.6 and Bartlett’s test of sphericity (*p *< 0.05) revealed that factor analysis was adequate [33]. Extraction was performed using the maximum variance (Varimax) approach to principal components analysis (PCA) [34]. A question-by-question deletion method was used to remove the items that didn’t measure up to the standard and expectations. The following conditions applied to deletion [35, 36]: (1) factor loadings <0.3; (2) the item simultaneously appears in two or more variables (Factor loadings were all greater than 0.4 and the difference was less than 0.2); (3) there were only 1–2 items in the factors.

#### 2.2.4 Content Validity Assessment

The relevance of each issue was also rated by experts in two rounds of Delphi expert consultations, ranging from 1 (completely irrelevant) to 4 (totally relevant). The number of experts who gave each item a score of 3 or 4 may be counted, and that number can then be divided by the total number of experts to determine the item-level content validity index (I-CVI) for each item [37]. The average of the entry’s I-CVI is used in the scale-level content validity index/averaging computation (S-CVI/Ave) [38]. An I-CVI of 0.78 and an S-CVI/Ave of 0.9 were regarded as outstanding content validity indicators [37].

#### 2.2.5 Reliability Assessment: Cronbach’s α and Item-to-Total Coefficient

To check the questionnaire’s internal consistency and reliability, we employed the Cronbach’s alpha coefficient, split-half reliability coefficient, and item-total correlation analysis. The following criteria are used to evaluate Cronbach’s α coefficient of internal consistency [39]: >0.7: passable, >0.8: decent, and >0.9: first-rate. Item-total correlation coefficient ≥0.4 suggested adequate scale homogeneity [40].

### 2.3 Data Analysis

For statistical analysis, SPSS version 24.0 for Windows (SPSS Inc., Chicago, IL, USA) was utilized. To assess the demographic characteristics, descriptive statistics (number, percentage, mean, and standard deviation) were used. The scale’s content validity (I-CVI and S-CVI/Ave), construct validity (EFA), reliability (Cronbach’s alpha and item-to-total coefficient), and item analysis were tested for validity and reliability. All *p*-values reflected bilateral probability, and the test level was set at 0.05.

### 2.4 Ethics

The Medical Ethics Committee of the Tongji Medical College, Huazhong University of Science & Technology, China, approved this study and it was carried out in compliance with the Declaration of Helsinki [41] (approval number: TJ-IRB20191221). Prior to conducting individual interviews and questionnaire surveys, informed consent was sought from each patient. Surveys were completed anonymously, all information gathered was strictly confidential, and the results were used solely for this study.

## 3. Results

### 3.1 Sample Characteristics

In all, 160 questionnaires were collected. The average patient age was 55.04 ± 11.68 years, and 76.9% of the patients were male. The majority of the patients were married (94.4%), had a junior high school education (32.5%), lived in an urban location (58.8%), lived with their spouse (86.9%), were employed (41.9%), had a monthly income <3000 (CNY) (45.0%), had a type B aortic dissection (67.5%), and had normal weight (47.5%). More than half of the patients had new rural cooperative medical insurance. Thoracic endovascular aortic repair (TEVAR) was done in 112 (70.0%) cases and surgery in 48 (30.0%). The length of stay of patients with aortic dissections of types A and B were 21.0 (18.0–29.0) days and 8.0 (4.0–14.0) days, respectively. Ninety-seven patients (60.6%) had a history of hypertension, and 82 patients (51.3%) had received previous medication. However, only a small proportion had a history of previous cardiac surgery (20.6%) and prior hospitalizations for AD (11.9%). Detailed demographic characteristics are presented in Table 1.

**Table 1.** **Demographic characteristics (N = 160)**.

Variable	n (%)	Variable	n (%)
Age (years), mean ± SD	55.04 ± 11.68	Medical fee payment method	
Gender		Self-payment	2 (1.3)
Male	123 (76.9)	Free medical care	0 (0.0)
Female	37 (23.1)	Urban medical insurance	65 (40.6)
Marital status		New rural cooperative medical insurance	92 (57.5)
Married	151 (94.4)	Commercial insurance	1 (0.6)
Divorced	1 (0.6)	Classification of AD	
Widowed	2 (1.2)	TAAD	52 (32.5)
Unmarried	6 (3.8)	TBAD	108 (67.5)
Education		Type of operation	
Primary school and below	48 (30.0)	TEVAR	112 (70.0)
Junior high school	52 (32.5)	Surgical operation	48 (30.0)
Senior high school	32 (20.0)	Length of stay (days, IQR)	
University and above	28 (17.5)	TAAD	21.0 (18.0–29.0)
Place of residence		TBAD	8.0 (4.0–14.0)
Urban	94 (58.8)	BMI categories	
Rural	66 (41.2)	Normal weight	76 (47.5)
Living situation		Overweight	54 (33.8)
Alone	6 (3.8)	Obese	30 (18.7)
Living with spouse	139 (86.9)	Smoking history	67 (41.9)
Living with children	62 (38.8)	Drinking history	62 (38.8)
Living with parents	13 (8.1)	Past history	
Occupational status		Hypertension	97 (60.6)
Unemployed	38 (23.7)	Kidney disease	6 (3.8)
Employed	67 (41.9)	Coronary heart disease	9 (5.6)
Retired	55 (34.4)	Cerebral infarction	2 (1.3)
Monthly income (CNY)		Myocardial infarction	1 (0.6)
<3000	72 (45.0)	Other	14 (8.8)
3000–5000	47 (29.4)	Previous medication history	82 (51.3)
>5000	41 (25.6)	History of previous cardiac surgery	33 (20.6)
		Prior hospitalizations for AD	19 (11.9)

Note: Continuous variables are reported as means ± standard deviations; counts are presented as the counts (percentage); non-normally distributed data presented as median (IQR, interquartile range). AD, aortic dissection; TAAD, type A aortic dissection; TBAD, type B aortic dissection; BMI, body-mass index. Weight divided by height squared was used to compute BMI, which was then categorized according to Chinese reference standards as underweight (BMI <18.5), normal (18.5–23.9), overweight (24–27.9), and obese (≥28). TEVAR, thoracic endovascular aortic repair.

### 3.2 Results of Delphi Expert Consultation

Eighty-three items were first created for the EABIQ using the findings of 24 AD patients’ combined semi-structured interviews. Eighteen questions were delivered in each of the two rounds of the Delphi polls. Eighteen questionnaires were returned with a 100% response rate in the first round of the Delphi technique. For the 18 specialists, 13 women and 5 males, the ages ranged from 40 to 58 years, with the average of 47.44 ± 5.06 years. The average years of professional experience was 23.61 ± 7.73 years. Regarding the experts’ respective titles, 55.6% (10/18) were senior vice title, and 44.4% (8/18) were senior title. Fifty-four items were ultimately accepted. Experts deemed an additional 29 items to be irrelevant or to not fulfill the standards for a consensus, thus they were removed. There were 16 experts in the second-round survey who were also in the experts’ group in the first round. However, no opinions were presented. The Kendall’s coefficient of concordance (W) was 0.205 (*p *< 0.01) and 0.333 (*p *< 0.01), respectively, for the two rounds of the Delphi consultation, and the Cr was found to be 0.842 and 0.856, respectively. This demonstrated that there was considerable agreement among the expert opinions. The results of the second round showed more synchronization than those of the previous round. The latest questionnaire contained 66 items.

### 3.3 Item Analysis

The findings of item selection based on the sample of 160 patients are displayed in **Supplementary Table 1**. Only item J6 was excluded as it did not meet five of the six-screening standard.

### 3.4 Validity Analysis Assessment

#### 3.4.1 Construct Validity Assessment

**Fig. 1.**
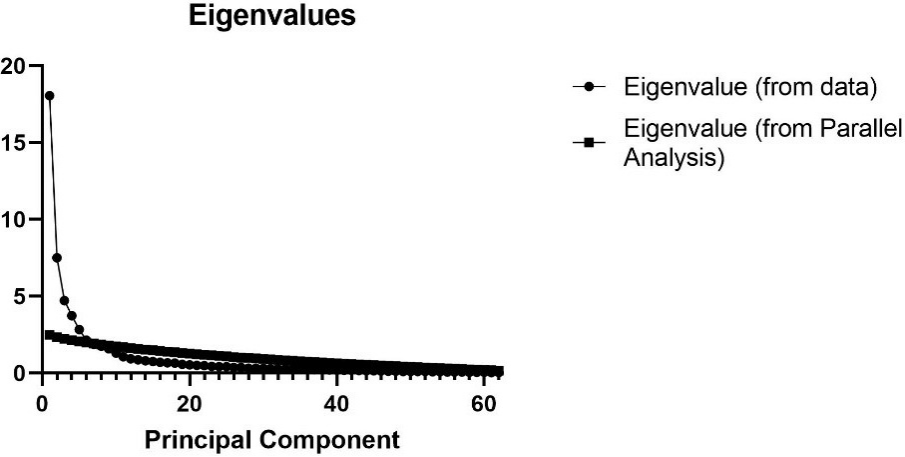
**Scree plot for the 62-item Exercise Attitudes and Behavioral Intentions Questionnaire (EABIQ) (n = 160)**.

EFA was conducted on 65 items of the EABIQ. The analysis process adopted an item-by-item deletion method to obtain an effective interpretation of the scale structure. Items D8 and E4 were deleted because it had multiple heavy loads in two factors, and the load value was close. Only item D7 existed alone in a factor and did not meet the criteria, so it was excluded. Eventually, 62 items were retained. The results of EFA showed that KMO was found to be 0.870, and the Bartlett sphericity test yielded the value *$X^{2}$* = 9098.663 (df = 1891, *p *< 0.001). These results demonstrated that the scale’s items were appropriate for factor analysis. 11 common components with eigenvalues larger than 1 were identified by exploratory factor analysis, and the scale’s variance explained cumulatively rate was 75.216 percent. The scree plot in Fig. 1 demonstrates that eigenvalues gradually declined after component 11, demonstrating that the scale’s EFA results were largely consistent with the proposed structures. There were eleven sub-dimensions with the following names: possibility of risk (6 items), severity of risk (5 items), controllability of risk (4 items), positive outcome expectations (6 items), negative outcome expectations (5 items), behavior intentions (7 items), implementation intention (including action planning and coping planning, 9 items), action self-efficacy (5 items), maintaining self-efficacy (7 items), recovery self-efficacy (3 items) and social support (5 items). For details see **Supplementary Table 2**.

#### 3.4.2 Content Validity

The I-CVI for the EABIQ varied from 0.813 to 1.000 after two rounds of the modified Delphi survey, and the S-CVI/Ave was 0.934.

#### 3.4.3 Reliability of the Scale and Correlation Coefficient from Item to Total

The Cronbach’s alpha for the entire scale was 0.929, and it ranged from 0.835 to 0.965 for each domain. For the full scale, the split-half dependability coefficient was 0.960 and varied from 0.844 to 0.962 for each domain. The detailed results were shown in Table 2. The correlation coefficient between each dimension of the scale and the overall scale was between 0.405 to 0.785, and all the *p*-values were less than 0.05.

**Table 2.** **Reliability metrics Cronbach’s alpha and Split-half dependability coefficients**.

Domains	Cronbach’s alpha coefficient	Split-half reliability coefficient
Possibility of risk	0.931	0.932
Severity of risk	0.887	0.859
Controllability of risk	0.873	0.844
Positive outcome expectations	0.835	0.849
Negative outcome expectations	0.855	0.875
Behavior intentions	0.861	0.847
Implementation intention	0.965	0.947
Action self-efficacy	0.943	0.951
Maintaining self-efficacy	0.961	0.962
Recovery self-efficacy	0.863	0.865
Social support	0.916	0.926
The total scale	0.929	0.960

## 4. Discussion

To our knowledge, this is the first survey to examine AD survivors’ views on exercise and behavior intentions. The theoretical framework of this study was based on the work of Schwarzer [26]. We developed the scale as a result of a thorough procedure including focus groups, interviews, expert advice, and quantitative psychometric testing. Eventually, 62 items were retained. According to the HAPA theory put forward by Schwarzer in 1992 [26], combined with the results of EFA, we divided the questionnaire into 11 dimensions, including possibility of risk, severity of risk, controllability of risk, positive outcome expectations, negative outcome expectations, behavior intentions, implementation intention, action self-efficacy, maintaining self-efficacy, recovery self-efficacy and social support. The results of EFA showed that action planning and coping planning were in the same dimension, so we combined the two into one dimension (implementation intention). In addition, in contrast from the HAPA theory, we found that social support was a very important factor for the persistence of exercise behavior in the interview process [19], so we added the dimension of social support to measure EABIQ. The modified questionnaire items were clear and easy to understand following a pilot study and formal investigation. With the help and guidance of researchers, the patients spent about 15–20 minutes to complete the questionnaire, which was acceptable to the researchers. This questionnaire enabled us to have a more comprehensive understanding of the attitudes, intentions and influencing factors of patients with AD towards exercise, to provide personalized exercise guidance and develop programs for patients with different psychological characteristics.

This study included a questionnaire survey involving 160 AD patients. Four methods were used for item analysis. Only item J6 did not meet the standard and was excluded after expert discussion. To guarantee the uniformity of the scale’s objectives. D7, D8, and E4 were eliminated by EFA, and the final 11 common factors’ total variance contribution rate was 75.216 percent. Each scale dimension’s correlation coefficient ranged from 0.405 to 0.785, suggesting adequate scale homogeneity. The Cronbach’s alpha for the entire scale was 0.929, and the alpha for each domain ranged from 0.835 to 0.965. The split-half reliability coefficient for the entire scale was 0.960, as well as the split-half reliability coefficient for each domain ranged from 0.844 to 0.962, indicating that the scale has good internal consistency and reliability. The questions were added to and adjusted following the Delphi survey’s two iterations; the I-CVI for the EABIQ varied from 0.813 to 1.000, and the S-CVI/Ave was 0.934. This showed that the content and distribution of each item were reasonable and highly recognized by experts [42]. By comparing two time points separated by two weeks, the Pearson’s correlation coefficient was used to determine the test-retest reliability [43]. However, because the patient’s condition was relatively stable at the time of the questionnaire survey, and most had been discharged when asked again two weeks later, retest reliability was not employed in this survey.

## 5. Limitations

This new instrument demonstrated satisfactory psychometric properties for measuring exercise attitudes and behavior intentions for AD patients. This study has several limitations. The participants were all from Wuhan, China’s third-class Class A hospital. Patients with type B aortic dissection were significantly greater than those with type A aortic dissection. These findings may not be representative of all Chinese patients. Future studies will need to be conducted in multiple centers, with a larger and more diverse patient population to further evaluate the adaptability of the questionnaire. Furthermore, the questionnaire involves multiple dimensions of psychological measurements, but the relationship and interaction between various dimensions are not clear, which needs to be further verified in larger trials.

## 6. Conclusions

Promoting the rehabilitation of patients with AD and improving the quality of life after surgery are crucial in these patients. This study used a 62-item questionnaire based on the HAPA theory to understand the attitudes and behavior intentions of AD patients toward exercise, which was found to have good reliability and validity. The questionnaire is not only a judgment tool to measure the exercise behavior for AD patients, but also provides a basis for the medical staff to provide phased and individualized intervention programs, so as to better promote enhanced and sustained recovery for AD patients.

## Supplementary Material

Supplementary material associated with this article can be found, in the online version, at https://doi.org/10.31083/j.rcm2310337.
