# Supplementary material for: Developing an Exercise Attitudes and Behavior Intentions Questionnaire for Survivors of Aortic Dissection: An Exploratory Factor Analysis
Source: Rev Cardiovasc Med. 2022 Oct 11;23(10):337. doi: 10.31083/j.rcm2310337 (PMC11267318; doi:10.31083/j.rcm2310337)
Supplement: Supplementary file 1 [file 2153-8174-23-10-337-s1.docx]

Supplementary Table 1. Item analysis of the Exercise Attitudes and Behavioral Intentions Questionnaire for aortic dissection survivors.

| Item | CR | Item-to-total correlation coefficient (r) | The correlation coefficient between the modified item and the total score | Cronbach's α if item deleted | Common value | Factor loading |
| --- | --- | --- | --- | --- | --- | --- |
| Possibility of risk |  |  |  |  |  |  |
| A1. The disease will recur after operation | 18.909 | 0.800^**^ | 0.725 | ↓ | 0.648 | 0.805 |
| A2. Respiratory function problems such as dyspnea and reduced lung capacity will occur early after operation | 21.881 | 0.898^**^ | 0.849 | ↓ | 0.815 | 0.903 |
| A3. In the long term after operation, there will be cardiopulmonary function problems such as decreased exercise tolerance | 22.687 | 0.904^**^ | 0.859 | ↓ | 0.825 | 0.908 |
| A4. Negative emotions such as anxiety and depression will occur after operation | 17.921 | 0.843^**^ | 0.766 | ↓ | 0.698 | 0.835 |
| A5. There will be sleep problems after operation | 17.115 | 0.835^**^ | 0.755 | ↓ | 0.684 | 0.827 |
| A6. Poor blood pressure control is easy to worsen the condition after operation | 24.223 | 0.894^**^ | 0.840 | ↓ | 0.806 | 0.898 |
| Severity of risk |  |  |  |  |  |  |
| B1. Deterioration will lead to other cardiovascular diseases | 19.276 | 0.862^**^ | 0.772 | ↓ | 0.774 | 0.880 |
| B2. The deterioration of the disease will affect my life span | 21.617 | 0.899^**^ | 0.847 | ↓ | 0.842 | 0.918 |
| B3. Illness will influence my quality of life after discharge | 27.382 | 0.900** | 0.829 | ↓ | 0.830 | 0.911 |
| B4. The aggravation of the disease will affect the life and work of the family | 22.861 | 0.897** | 0.829 | ↓ | 0.830 | 0.911 |
| B5. The aggravation of the disease will increase the economic burden of the family | 10.500 | 0.623** | 0.425 | ↑ | 0.302 | 0.550 |
| Controllability of risk |  |  |  |  |  |  |
| C1. Regular exercise helps to control the progress of the disease | 8.001 | 0.858** | 0.743 | ↓ | 0.753 | 0.868 |
| C2. Regular exercise helps to prolong my life | 12.515 | 0.878** | 0.782 | ↓ | 0.789 | 0.888 |
| C3. Regular exercise helps to control the occurrence or progression of cardiopulmonary function problems (such as dyspnea, decreased exercise tolerance, etc.) | 10.020 | 0.872** | 0.765 | ↓ | 0.766 | 0.875 |
| C4. Regular exercise can help delay the development of other cardiovascular diseases (such as hypertension, coronary heart disease, etc.) | 3.367 | 0.803** | 0.635 | ↑ | 0.611 | 0.781 |
| Positive outcome expectations |  |  |  |  |  |  |
| D1. It will help me increase exercise tolerance and improve cardiopulmonary function | 4.783 | 0.671** | 0.573 | ↓ | 0.546 | 0.758 |
| D2. It will help me maintain good blood pressure | 8.324 | 0.702** | 0.609 | ↓ | 0.534 | 0.739 |
| D3. It will keep my weight in a healthy range | 3.692 | 0.671** | 0.572 | ↓ | 0.531 | 0.730 |
| D4. It will help me form healthy living habits | 4.381 | 0.702** | 0.596 | ↓ | 0.575 | 0.728 |
| D5. It will help me reduce anxiety, depression and other negative emotions | 4.575 | 0.644** | 0.536 | ↓ | 0.460 | 0.724 |
| D6. It will help me improve my sleep quality | 6.216 | 0.673** | 0.572 | ↓ | 0.525 | 0.678 |
| D7. Others will admire my perseverance and patience | 12.153 | 0.643** | 0.357 | ↑ | 0.208 | 0.577 |
| D8. It will increase my self-confidence | 3.103 | 0.621** | 0.468 | ↓ | 0.333 | 0.456 |
| Negative outcome expectations |  |  |  |  |  |  |
| E1. Pay will be greater than reward | 10.302 | 0.816** | 0.727 | ↓ | 0.689 | 0.830 |
| E2. It will take some time and energy | 9.677 | 0.762** | 0.620 | ↓ | 0.533 | 0.730 |
| E3. It will increase the economic burden | 7.912 | 0.713** | 0.575 | ↓ | 0.472 | 0.687 |
| E4. Accidents will happen | 10.142 | 0.758** | 0.652 | ↓ | 0.603 | 0.776 |
| E5. It will increase family concerns | 11.519 | 0.836** | 0.756 | ↓ | 0.723 | 0.850 |
| E6. Failure to achieve the expected goal of exercise will lead to psychological loss | 11.599 | 0.836** | 0.757 | ↓ | 0.718 | 0.848 |
| Behavioral intentions |  |  |  |  |  |  |
| F1. Consult and learn about the knowledge of exercise | 16.628 | 0.748** | 0.624 | ↓ | 0.539 | 0.734 |
| F2. Start exercising with appropriate frequency and intensity | 11.920 | 0.838** | 0.753 | ↓ | 0.703 | 0.838 |
| F3. Change bad living habits | 10.630 | 0.829** | 0.742 | ↓ | 0.689 | 0.830 |
| F4. Smoking cessation and limited alcohol intake | 7.104 | 0.687** | 0.554 | ↓ | 0.457 | 0.676 |
| F5. Start weight management | 8.774 | 0.740** | 0.633 | ↓ | 0.545 | 0.738 |
| F6. Monitor blood pressure regularly | 8.921 | 0.723** | 0.622 | ↓ | 0.541 | 0.735 |
| F7. Regularly review the progress of the disease | 6.111 | 0.597** | 0.502 | ↓ | 0.379 | 0.615 |
| Action planning |  |  |  |  |  |  |
| G1. Plan about what time to start exercising | 28.989 | 0.920** | 0.886 | ↓ | 0.846 | 0.920 |
| G2. Plan about where to exercise | 24.493 | 0.866** | 0.828 | ↓ | 0.764 | 0.874 |
| G3. Plan about the length of the exercise | 28.104 | 0.912** | 0.875 | ↓ | 0.832 | 0.912 |
| G4. Plan about how often to exercise | 26.700 | 0.907** | 0.874 | ↓ | 0.829 | 0.910 |
| G5. Plan about who to exercise with | 25.889 | 0.894** | 0.848 | ↓ | 0.789 | 0.888 |
| G6. Supervisor of the implementation of the exercise program | 24.783 | 0.876** | 0.828 | ↓ | 0.763 | 0.873 |
| G7. Specific forms of exercise | 24.756 | 0.878** | 0.831 | ↓ | 0.768 | 0.876 |
| Coping planning |  |  |  |  |  |  |
| H1. If there is a situation that interferes with the exercise program, I have a way to deal with it | 25.783 | 0.926** | 0.712 | __ | 0.856 | 0.925 |
| H2. If discomfort occurs during exercise, I have ways to deal with it | 24.104 | 0.924** | 0.712 | __ | 0.856 | 0.925 |
| Action self-efficacy |  |  |  |  |  |  |
| I1. I have to learn a lot about rehabilitation exercise | 15.842 | 0.914** | 0.866 | ↓ | 0.840 | 0.917 |
| I2. I have to reconsider my view of exercise | 18.441 | 0.900** | 0.839 | ↓ | 0.806 | 0.898 |
| I3. I think it's hard to do exercise | 14.569 | 0.882** | 0.813 | ↓ | 0.776 | 0.881 |
| I4. I think it's hard to keep exercising for a long time | 21.731 | 0.912** | 0.859 | ↓ | 0.831 | 0.912 |
| I5. I need to start doing an exercise program | 17.463 | 0.911** | 0.857 | ↓ | 0.830 | 0.911 |
| Maintaining self-efficacy |  |  |  |  |  |  |
| J1. My blood pressure doesn't improve immediately | 26.373 | 0.904** | 0.868 | ↓ | 0.832 | 0.912 |
| J2. When the effect of disease improvement is not obvious | 24.085 | 0.888** | 0.847 | ↓ | 0.802 | 0.895 |
| J3. I feel depressed | 24.706 | 0.907** | 0.873 | ↓ | 0.841 | 0.917 |
| J4. I feel very tired | 24.635 | 0.907** | 0.874 | ↓ | 0.842 | 0.918 |
| J5. I'm under a lot of pressure | 21.925 | 0.897** | 0.862 | ↓ | 0.826 | 0.909 |
| J6. My companions and family don't support me | 26.244 | 0.905** | 0.870 | ↓ | 0.835 | 0.914 |
| J7. Neither my companions nor my family exercised with me | 16.169 | 0.827** | 0.773 | ↓ | 0.688 | 0.829 |
| J8. I feel mental tension | 9.687 | 0.398** | 0.259 | ↑ | 0.095 | 0.308 |
| Recovery self-efficacy |  |  |  |  |  |  |
| K1. Occasionally, I changed my exercise plan several times | 12.549 | 0.879** | 0.728 | ↓ | 0.772 | 0.879 |
| K2. No exercise program for one month | 15.437 | 0.898** | 0.752 | ↓ | 0.798 | 0.893 |
| K3. No exercise program for several months | 16.830 | 0.882** | 0.743 | ↓ | 0.788 | 0.888 |
| Social support |  |  |  |  |  |  |
| L1. Friends or family members will work with me to develop an exercise plan | 25.133 | 0.894** | 0.832 | ↓ | 0.805 | 0.897 |
| L2. Friends or family will work out with me | 25.975 | 0.900** | 0.826 | ↓ | 0.800 | 0.894 |
| L3. Friends or family members will encourage me to stick to the exercise program | 15.117 | 0.890** | 0.820 | ↓ | 0.795 | 0.891 |
| L4. Friends or family members will supervise me to complete the exercise program | 16.730 | 0.889** | 0.817 | ↓ | 0.785 | 0.886 |
| L5. I will get the help and guidance of medical professionals | 10.708 | 0.753** | 0.648 | ↑ | 0.574 | 0.758 |

Note: CR, critical ration. The text highlighted in gray indicates that it does not meet the screening criteria. "-" means that there is no measurement because only two items cannot be verified. “*” means P < 0.01.

Supplementary Table 2. Exploratory factor analysis of Exercise Attitudes and Behavioral Intentions Questionnaire for aortic dissection survivors.

| Item | Factor loadings | | | | | | | | | | |
| --- | --- | --- | --- | --- | --- | --- | --- | --- | --- | --- | --- |
|  | 1 | 2 | 3 | 4 | 5 | 6 | 7 | 8 | 9 | 10 | 11 |
| Action planning and Coping planning |  |  |  |  |  |  |  |  |  |  |  |
| G1. Plan about what time to start exercising | 0.865 |  |  |  |  |  |  |  |  |  |  |
| G3. Plan about the length of the exercise | 0.854 |  |  |  |  |  |  |  |  |  |  |
| G4. Plan about how often to exercise | 0.846 |  |  |  |  |  |  |  |  |  |  |
| G2. Plan about where to exercise | 0.826 |  |  |  |  |  |  |  |  |  |  |
| G5. Plan about who to exercise with | 0.813 |  |  |  |  |  |  |  |  |  |  |
| G7. Specific forms of exercise | 0.801 |  |  |  |  |  |  |  |  |  |  |
| H1. If there is a situation that interferes with the exercise program, I have a way to deal with it | 0.793 |  |  |  |  |  |  |  |  |  |  |
| G6. Supervisor of the implementation of the exercise program | 0.774 |  |  |  |  |  |  |  |  |  |  |
| H2. If discomfort occurs during exercise, I have ways to deal with it | 0.747 |  |  |  |  |  |  |  |  |  |  |
| Maintaining self-efficacy |  |  |  |  |  |  |  |  |  |  |  |
| J3. I feel depressed |  | 0.899 |  |  |  |  |  |  |  |  |  |
| J4. I feel very tired |  | 0.881 |  |  |  |  |  |  |  |  |  |
| J6. My companions and family don't support me |  | 0.856 |  |  |  |  |  |  |  |  |  |
| J2. When the effect of disease improvement is not obvious |  | 0.855 |  |  |  |  |  |  |  |  |  |
| J5. I'm under a lot of pressure |  | 0.846 |  |  |  |  |  |  |  |  |  |
| J1. My blood pressure doesn't improve immediately |  | 0.826 |  |  |  |  |  |  |  |  |  |
| J7. Neither my companions nor my family exercised with me |  | 0.680 |  |  |  |  |  |  |  |  |  |
| Possibility of risk |  |  |  |  |  |  |  |  |  |  |  |
| A3. In the long term after operation, there will be cardiopulmonary function problems such as decreased exercise tolerance |  |  | 0.883 |  |  |  |  |  |  |  |  |
| A6. Poor blood pressure control is easy to worsen the condition after operation |  |  | 0.883 |  |  |  |  |  |  |  |  |
| A2. Respiratory function problems such as dyspnea and reduced lung capacity will occur early after operation |  |  | 0.879 |  |  |  |  |  |  |  |  |
| A1. The disease will recur after operation |  |  | 0.799 |  |  |  |  |  |  |  |  |
| A5. There will be sleep problems after operation |  |  | 0.789 |  |  |  |  |  |  |  |  |
| A4. Negative emotions such as anxiety and depression will occur after operation |  |  | 0.774 |  |  |  |  |  |  |  |  |
| Action self-efficacy |  |  |  |  |  |  |  |  |  |  |  |
| I5. I need to start doing an exercise program |  |  |  | 0.840 |  |  |  |  |  |  |  |
| I1. I have to learn a lot about rehabilitation exercise |  |  |  | 0.833 |  |  |  |  |  |  |  |
| I4. I think it's hard to keep exercising for a long time |  |  |  | 0.766 |  |  |  |  |  |  |  |
| I3. I think it's hard to do exercise |  |  |  | 0.762 |  |  |  |  |  |  |  |
| I2. I have to reconsider my view of exercise |  |  |  | 0.750 |  |  |  |  |  |  |  |
| Social support |  |  |  |  |  |  |  |  |  |  |  |
| L2. Friends or family will work out with me |  |  |  |  | 0.787 |  |  |  |  |  |  |
| L3. Friends or family members will encourage me to stick to the exercise program |  |  |  |  | 0.776 |  |  |  |  |  |  |
| L1. Friends or family members will work with me to develop an exercise plan |  |  |  |  | 0.776 |  |  |  |  |  |  |
| L4. Friends or family members will supervise me to complete the exercise program |  |  |  |  | 0.775 |  |  |  |  |  |  |
| L5. I will get the help and guidance of medical professionals |  |  |  |  | 0.665 |  |  |  |  |  |  |
| Positive outcome expectations |  |  |  |  |  |  |  |  |  |  |  |
| D1. It will help me increase exercise tolerance and improve cardiopulmonary function |  |  |  |  |  | 0.758 |  |  |  |  |  |
| D2. It will help me maintain good blood pressure |  |  |  |  |  | 0.756 |  |  |  |  |  |
| D3. It will keep my weight in a healthy range |  |  |  |  |  | 0.725 |  |  |  |  |  |
| D5. It will help me reduce anxiety, depression and other negative emotions |  |  |  |  |  | 0.694 |  |  |  |  |  |
| D6. It will help me improve my sleep quality |  |  |  |  |  | 0.674 |  |  |  |  |  |
| D4. It will help me form healthy living habits |  |  |  |  |  | 0.664 |  |  |  |  |  |
| Negative outcome expectations |  |  |  |  |  |  |  |  |  |  |  |
| E3. It will increase the economic burden |  |  |  |  |  |  | 0.774 |  |  |  |  |
| E6. Failure to achieve the expected goal of exercise will lead to psychological loss |  |  |  |  |  |  | 0.734 |  |  |  |  |
| E5. It will increase family concerns |  |  |  |  |  |  | 0.729 |  |  |  |  |
| E1. Pay will be greater than reward |  |  |  |  |  |  | 0.712 |  |  |  |  |
| E2. It will take some time and energy |  |  |  |  |  |  | 0.647 |  |  |  |  |
| Controllability of risk |  |  |  |  |  |  |  |  |  |  |  |
| C3. Regular exercise helps to control the occurrence or progression of cardiopulmonary function problems (such as dyspnea, decreased exercise tolerance, etc.) |  |  |  |  |  |  |  | 0.824 |  |  |  |
| C4. Regular exercise can help delay the development of other cardiovascular diseases (such as hypertension, coronary heart disease, etc.) |  |  |  |  |  |  |  | 0.808 |  |  |  |
| C1. Regular exercise helps to control the progress of the disease |  |  |  |  |  |  |  | 0.750 |  |  |  |
| C2. Regular exercise helps to prolong my life |  |  |  |  |  |  |  | 0.675 |  |  |  |
| Behavioral intentions |  |  |  |  |  |  |  |  |  |  |  |
| F7. Regularly review the progress of the disease |  |  |  |  |  |  |  |  | 0.727 |  |  |
| F4. Smoking cessation and limited alcohol intake |  |  |  |  |  |  |  |  | 0.680 |  |  |
| F6. Monitor blood pressure regularly |  |  |  |  |  |  |  |  | 0.654 |  |  |
| F5. Start weight management |  |  |  |  |  |  |  |  | 0.565 |  |  |
| F1. Consult and learn about the kowledge of exercise |  |  |  |  |  |  |  |  | 0.539 |  |  |
| F3. Change bad living habits |  |  |  |  |  |  |  |  | 0.442 |  |  |
| F2. Start exercising with appropriate frequency and intensity |  |  |  |  |  |  |  |  | 0.401 |  |  |
| Severity of risk |  |  |  |  |  |  |  |  |  |  |  |
| B2. The deterioration of the disease will affect my life span |  |  |  |  |  |  |  |  |  | 0.711 |  |
| B1. Deterioration will lead to other cardiovascular diseases |  |  |  |  |  |  |  |  |  | 0.690 |  |
| B4. The aggravation of the disease will affect the life and work of the family |  |  |  |  |  |  |  |  |  | 0.664 |  |
| B3. Illness will influence my quality of life after discharge |  |  |  |  |  |  |  |  |  | 0.633 |  |
| B5. The aggravation of the disease will increase the economic burden of the family |  |  |  |  |  |  |  |  |  | 0.585 |  |
| Recovery self-efficacy |  |  |  |  |  |  |  |  |  |  |  |
| K1. Occasionally, I changed my exercise plan several times |  |  |  |  |  |  |  |  |  |  | 0.639 |
| K2. No exercise program for one month |  |  |  |  |  |  |  |  |  |  | 0.575 |
| K3. No exercise program for several months |  |  |  |  |  |  |  |  |  |  | 0.506 |
| Characteristic root | 8.225 | 7.222 | 6.119 | 4.260 | 3.815 | 3.612 | 3.244 | 3.184 | 3.022 | 2.456 | 1.476 |
| Percentage of variance explained (%) | 13.265 | 11.648 | 9.870 | 6.870 | 6.153 | 5.825 | 5.232 | 5.136 | 4.874 | 3.962 | 2.380 |
| Cumulative percentage of variance explained (%) ^a^ | 13.265 | 24.914 | 34.783 | 41.654 | 47.807 | 53.632 | 58.864 | 64.000 | 68.874 | 72.836 | 75.216 |

a Extraction method: Principal Components Analysis (PCA), rotation with maximum variance (Varimax) method. Rotation converged in nine iterations.
